# Host–guest supra­molecular inter­actions between a resorcinarene-based cavitand bearing a –COOH moiety and acetic acid

**DOI:** 10.1107/S2056989019002512

**Published:** 2019-02-22

**Authors:** Alessandro Pedrini

**Affiliations:** a Department of Materials Science, University of Milan - Bicocca, Via Cozzi 55, 20125 Milan, Italy

**Keywords:** crystal structure, resorcinarene-based cavitands, acetic acid, host–guest complexes, hydrogen bonding, offset π–π inter­actions

## Abstract

The cavitand 5,11,17,23-tetra­methyl-4,24:6,10:12,16:18,22-tetra­kis­(methyl­enedi­oxy)resorcin[4]arene functionalized at the upper rim with a carb­oxy­lic acid group, **CavCOOH-in**, of chemical formula C_37_H_32_O_10_, was synthesized in order to study its supra­molecular inter­actions with acetic acid in the solid state. Crystals suitable for X-ray diffraction analysis were obtained by slow evaporation of a di­chloro­methane–acetone solution of **CavCOOH-in**, to which glacial acetic acid had been added. The resulting compound, C_37_H_32_O_10_·2C_2_H_4_O_2_ (**1**) crystallizes in the space group *P*  and its asymmetric unit consists of one mol­ecule of cavitand and two mol­ecules of acetic acid, one of which is encapsulated inside the aromatic cavity and disordered over two positions with a refined occupancy ratio of 0.344 (4):0.656 (4). The guest inter­acts with the host primarily through its methyl group, which (in both orientations) forms C—H⋯π inter­actions with the benzene rings of the cavitand. The crystal structure of **1** is dominated by O—H⋯O and C—H⋯O hydrogen bonding due to the presence of acetic acid and of the carb­oxy­lic group functionalizing the upper rim. Further stabilization is provided by offset π–π stacking inter­actions between the aromatic walls of adjacent cavitands [inter­centroid distance = 3.573 (1) Å].

## Abstract 2

Acetic acid is a by-product of peracetic acid with acute irritant properties when present in air. The cavitand 5,11,17,23-tetra­methyl-4,24:6,10:12,16:18,22-tetra­kis­(methyl­enedi­oxy)resorcin[4]arene functionalized at the upper rim with a carb­oxy­lic acid group, **CavCOOH-in**, was synthesized and crystallized with acetic acid to evaluate its mol­ecular recognition properties towards this analyte in the solid state.

## Chemical context   

Aseptic packaging utilizes hydrogen peroxide or peracetic acid for the sterilization of the packaging material and machines, enabling the introduction of beverages without additional thermal stress or added preservatives. By-products of peracetic acid are hydrogen peroxide and acetic acid. Acetic acid has acute irritant properties [The National Institute for Occupational Safety and Health NIOSH (https://www.cdc.gov/niosh/index.htm)] and its exposure limit value has been set at 10 ppm TWA. It is therefore important to find an accurate method to measure acetic acid vapour in order to assess the environmental air quality. In the literature, only one example of the environmental monitoring of gaseous acetic acid has been reported (Yan *et al.*, 2014 ▸). In particular, the authors presented the use of a quartz crystal microbalance (QCM) sensor on which a polyaniline film for the environmental monitoring of acetic acid was electrochemically polymerized. In the past, the QCM approach has also been used in combination with resorcinarene-based cavitands for the mol­ecular recognition of short-chain linear alcohols (Melegari *et al.*, 2008 ▸), and for the detection of aromatic hydro­carbons in water (Giannetto *et al.*, 2018 ▸). Cavitands, bowl-shaped synthetic macrocycles (Cram, 1983 ▸), have been successfully employed as sensors at the solid–gas inter­face (Pinalli *et al.*, 2018 ▸; Tudisco *et al.*, 2016 ▸), and also as building blocks for crystal engineering (Pinalli *et al.*, 2016 ▸). In order to endow the preorganized cavity with hydrogen-bonding acceptor and donor properties, a tetra­methyl­eneresorcin[4]arene functionalized at the upper rim with a carb­oxy­lic acid group, **CavCOOH-in**, was synthesized as receptor for the recognition of acetic acid. Preliminary studies were then carried out in the solid state through X-ray diffraction methods on single crystals, to analyze the weak inter­actions responsible for the recognition event. In this context, we report herein and discuss the crystal and mol­ecular structure of the title complex of **CavCOOH-in** with acetic acid, compound **1**.
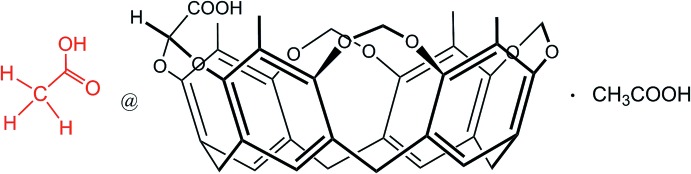


## Structural commentary   

**Figure 1:**
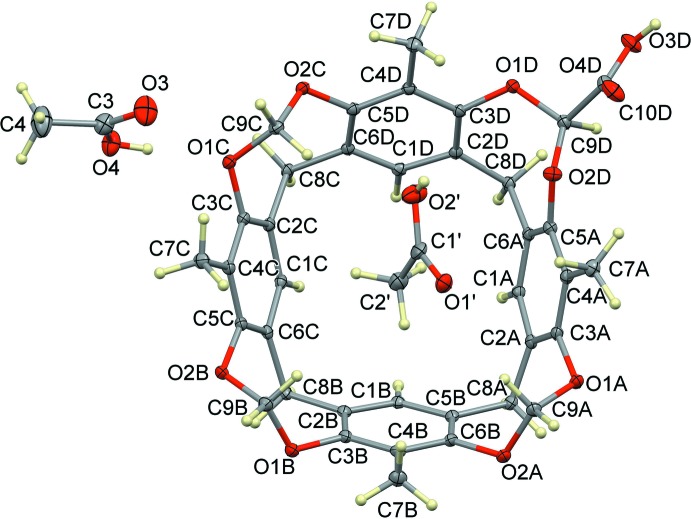
Top view of the mol­ecular structure of **1**, with the labelling scheme and displacement ellipsoids drawn at the 20% probability level. For clarity, only one of the two orientations for the disordered acetic acid mol­ecule inside the cavity is shown.

**Figure 2:**
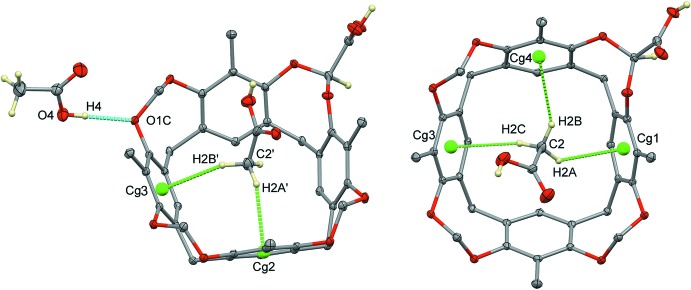
Left: view of the supra­molecular inter­actions (blue and green dotted lines) in **1** involving the acetic acid mol­ecules C1′/C2′/O1′/O2′ and C3/C4/O3/O4. Right: view of the supra­molecular inter­actions (green dotted lines) in **1** involving the acetic acid mol­ecule C1/C2/O1/O2.

**CavCOOH-in** is a tetra­methyl­eneresorcin[4]arene in which one of the four methyl­ene bridges at the upper rim is functionalized with a –COOH carb­oxy­lic unit. Following a previously published synthetic pathway (Daly *et al.*, 2007 ▸), two isomers can be obtained: CavCOOH-in and CavCOOH-out, depending whether the carb­oxy­lic group points inside or outside the cavity. The title compound is the isomer **CavCOOH-in**, as can be seen looking at the substituents on the carbon atom C9*D* in Fig. 1 ▸. The mol­ecular structure of the 1:1 host–guest complex between **CavCOOH-in** and acetic acid (**1**) is also shown in Fig. 1 ▸. Compound **1** crystallizes in the space group *P* 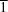 with two mol­ecules of acetic acid in the asymmetric unit, one encapsulated inside the aromatic cavity and disordered over two positions with occupancies of 0.344 (4) and 0.656 (4), respectively (C1′/C2′/O1′/O2′ and C1/C2/O1/O2) and one outside (C3/C4/O3/O4). The relevant supra­molecular inter­actions present in the asymmetric unit are shown in Fig. 2 ▸ and in Table 1 ▸. The acetic acid C3/C4/O3/O4 forms a hydrogen bond with the bridging resorcinol oxygen atom O1*C*, while the methyl group of the acetic acid held inside the cavity forms C—H⋯π inter­actions with the aromatic rings of the walls (see Table 1 ▸). The guest also forms a set of intra­molecular C—H⋯O inter­actions (in both orientations) involving the carb­oxy­lic oxygen atoms and the methyl and methyl­enic groups. Of the four methyl­ene bridges of the upper rim, three (atoms C9*A*, C9*B* and C9*C*, see Fig. 1 ▸) point inside the cavity, while C9*C* and its carb­oxy­lic substituent are distorted towards the outside (despite the isomer being **CavCOOH-in**), as can be seen from the C3—O1—C9—O2 torsion angles [C3*A*—O1*A*—C9*A*—O2*A* = 90.9 (2)°; C3*B*—O1*B*—C9*B*—O2*B* = 95.2 (2)°; C3*C*—O1*C*—C9*C*—O2*C* = 95.7 (2)°; C3*D*—O1*D*—C9*D*—O2*D* = −46.7 (3)°]. This is probably due to the hydrogen bonding in which the carb­oxy­lic acid C9*D*/C10*D*/O3*D*/O4*D* is involved with adjacent cavitands, as will be described in *Section 3*.

## Supra­molecular features   

**Figure 3:**
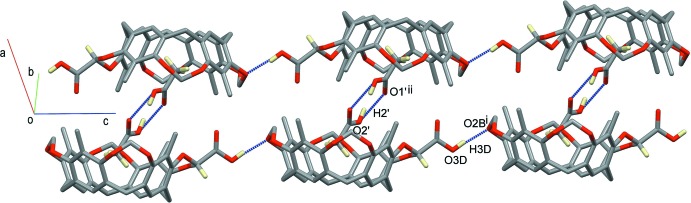
A view of the supra­molecular chain in the crystal structure of **1**, propagating along the *c*-axis direction. For clarity, only the H atoms involved in the formation of hydrogen bonds have been included [symmetry codes: (i) *x*, *y*, *z* + 1; (ii) −*x* + 2, −*y* + 1, −*z* + 1].

**Figure 4:**
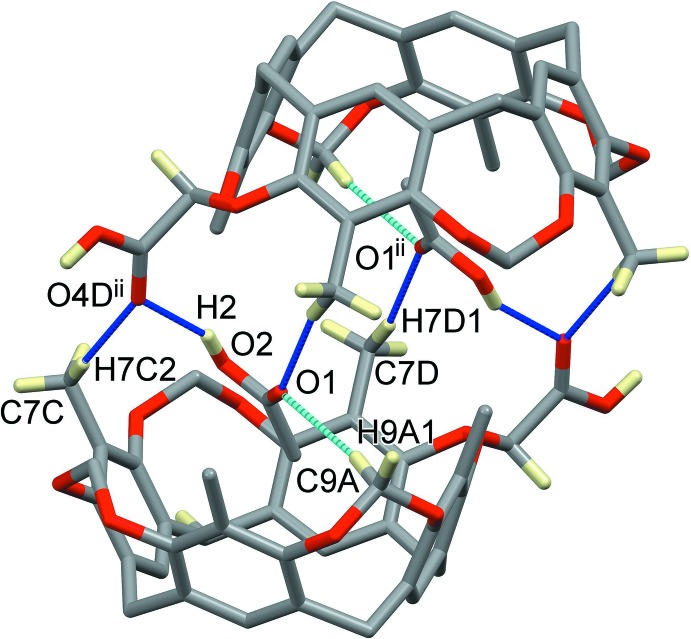
Intra- and inter­molecular contacts (cyan and blue dotted lines, respectively) involving the acetic acid guest in the orientation C1/C2/O1/O2. For clarity, only the H atoms involved in the formation of hydrogen bonds have been included [symmetry codes: (i) *x*, *y*, *z* + 1; (ii) −*x* + 2, −*y* + 1, −*z* + 1].

**Figure 5:**
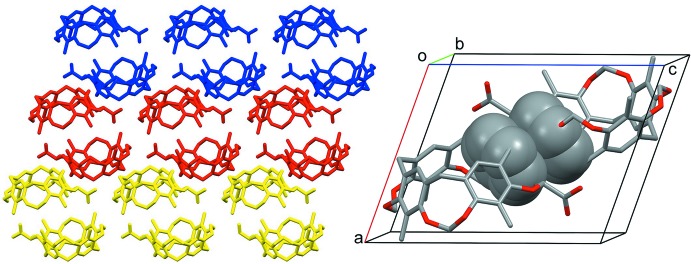
View of the three sets of ribbons (highlighted in blue, red and yellow) forming π–π stacking inter­actions involving pairs of inversion-related (−*x* + 1, −*y* + 1, −*z* + 1) aromatic rings, C1*A*–C6*A* (right).

While the main supra­molecular contacts at play for the encapsulation of acetic acid inside the cavitand are C—H⋯π inter­actions (Table 1 ▸), the crystal structure of **1** is dominated by hydrogen bonding. A chain which propagates along the *c*-axis direction is formed by strong O—H⋯O inter­actions involving the hydroxyl group O3*D*—H3*D* from the carb­oxy­lic acid at the methyl­ene bridge and the bridging resorcinol oxygen atom O2*B* ^i^ of an adjacent cavitand (Fig. 3 ▸ and Table 1 ▸). Pairs of chains form ribbons through the crystal, the cavitands facing one another, *via* supra­molecular inter­actions involving the acetic acid guest. In particular, C1′/C2′/O1′/O2′ forms a classical hydrogen-bonded inversion dimer with its symmetry-related analogue at −*x* + 2, −*y* + 1, −*z* + 1 (O2′—H2′⋯O1′; Fig. 3 ▸ and Table 1 ▸). When the acetic acid guest is in the other orientation, namely C1/C2/O1/O2, this dimer is not formed, but the guest acts as a hydrogen-bond donor with the hydroxyl group O2—H2 towards the oxygen atom O4*D* ^ii^ of the carb­oxy­lic acid at the methyl­ene bridge of an adjacent cavitand [symmetry code: (ii) −*x* + 2, −*y* + 1, −*z* + 1; see Fig. 4 ▸ and Table 1 ▸). On the other hand, atom O1 forms two C—H⋯O contacts, an inter­molecular one with a methyl group at the upper rim of a symmetry-related cavitand [C7*D-*–H7*D*1⋯O1^ii^] and an intra­molecular one with a methyl­ene bridge [C9*A*—H9*A*1⋯O1]. These sets of inter­actions are completed by another inter­molecular C—H⋯O hydrogen bond between methyl group C7*C*—H7*C*2 and the carboxyl oxygen atom O4*D* ^ii^. Finally, the ribbons (highlighted in blue, red and yellow in Fig. 5 ▸) form offset π–π stacking inter­actions involving pairs of inversion-related (−*x* + 1, −*y* + 1, −*z* + 1) C1*A–*C6*A* aromatic rings [Fig. 5 ▸ right-hand-side; centroid–centroid distance = 3.573 (1) Å; slippage = 1.338 Å].

## Database survey   

A resorcinarene-based cavitand in which one of the four methyl­enic bridges is functionalized with a carb­oxy­lic acid is unique to the present day. An isomer of the title compound (XIDLIG) and its analogue with four –C_5_H_11_ alkyl chains at the lower rim (XIDLEC) have been used to form supra­molecular complexes with di­methyl­methyl­phospho­nate, DMMP, a nerve-gas simulant bearing a P=O group (Daly *et al.*, 2007 ▸). XIDLIG and XIDLEC do not only differ from each other in the lower rim substituents, but also in the orientation of the –COOH group (outward and inward, respectively) with respect to the cavity. The presence of this group is pivotal in providing the cavity with a hydrogen-bond donor towards the P=O fragment of DMMP; when –COOH points inward, not only is this hydrogen bond formed, but DMMP enters the cavity with one of its methyl groups, forming C—H⋯π inter­actions with the aromatic walls of the cavitand. In the case of the title compound **1**, an acetic acid mol­ecule enters the cavity with the methyl group but the hydrogen bond is formed with another symmetry-related mol­ecule of acetic acid. The –COOH fragment on the methyl­ene bridge is hence free to hydrogen bond to the resorcinol oxygen atom of an adjacent cavitand, giving rise to the supra­molecular chain described in *Section 3*. A search in the Cambridge Structural Database (CSD, Version 5.38, update August 2018; Groom *et al.*, 2016 ▸) for a cavitand bearing a carb­oxy­lic acid moiety at the upper rim gave six hits other than XIDLIG and XIDLEC, namely compounds ILIJOC and ILIJUI (Kobayashi *et al.*, 2003 ▸), KAHMOV (Kobayashi *et al.*, 2000 ▸), LOPKEG (Kobayashi *et al.*, 1999 ▸), OSIYIA and OSIYOG (Aakeröy *et al.*, 2016 ▸). In all these structures, the –COOH moiety is employed to build supra­molecular architectures through hydrogen bonding. More precisely, in the case of ILIJOC and ILIJUI, a tetra­methyl­eneresorcin[4]arene functionalized with four carb­oxy­lic groups on the aromatic walls of the cavity (**A**) has been used to form a heterodimeric capsule in a rim-to-rim fashion through the formation of four hydrogen bonds with a tetra­(3-pyrid­yl)-cavitand. The previously cited cavitand **A** self-assembles into a one-dimensional chain (LOPKEG) or into dimeric capsules (KAHMOV) *via* hydrogen bonding with four 2-amino­pyrimidine mol­ecules. Similarly, OSIYIA and OSIYOG consist of supra­molecular self-assembled polymers or capsules between tetra­carb­oxy­lic acid functionalized cavitands and suitable *N*-heterocyclic linkers such as 4,4-bi­pyridine and 2-amino-5-bromo-4-chloro-6-methyl­pyrimidine.

## Synthesis and crystallization   

The synthesis of cavitand **CavCOOH-in** was carried out according to the procedure employed for the **CavCOOH-out** isomer (Daly *et al.*, 2007 ▸). ^1^H NMR spectra were obtained using a Bruker AMX-300 (300 MHz) spectrometer. All chemical shifts (δ) are reported in p.p.m. relative to the proton resonances resulting from incomplete deuteration of the NMR solvents. ^1^H NMR (CDCl_3_, 300 MHz) *d* = 1.91 (*s*, 6H, ArCH_3_), 2.01 (*s*, 6H, ArCH_3_), 3.23 (*m*, 4H, CH_eq_), 4.31 (*m*, 3H, O–CH_in_–O), 4.51 (*m*, 4H, CH_ax_), 5.85 (*m*, 3H, O–CH_out_–O,), 6.73 (*s*, 1H, CH_out_-COOH), 6.94 (*bs*, 4H, ArH).

Colourless crystals of the inclusion complex **1** were obtained by slow evaporation of a solution prepared by dissolving 0.005 mmol of the cavitand **CavCOOH-in** in 5 ml of a 1:1 di­chloro­methane and acetone solution, to which 1.1 µL (0.02 mmol) of glacial acetic acid were added.

## Refinement   

Crystal data, data collection and structure refinement details are summarized in Table 2 ▸. The H atoms bound to C and O were placed in calculated positions and refined isotropically using the riding model with C–H ranging from 0.95 to 0.99 Å, O—H = 0.84 Å and *U*_iso_(H) set to 1.2–1.5*U*_eq_(C/O), the only exception being atom H9*D*, which was located in a difference-Fourier map and refined freely. A DIFX instruction was employed to avoid a short H⋯H contact between atoms H9*D* and H8*D*1. Atoms O1 and O2 were refined using the EADP command. The acetic acid guest is disordered over two positions with a refined occupancy ratio of 0.344 (4):0.656 (4).

## Supplementary Material

Crystal structure: contains datablock(s) I, Global. DOI: 10.1107/S2056989019002512/su5481sup1.cif

Structure factors: contains datablock(s) I. DOI: 10.1107/S2056989019002512/su5481Isup2.hkl

CCDC reference: 1897735

Additional supporting information:  crystallographic information; 3D view; checkCIF report

## Figures and Tables

**Table 1:** Hydrogen-bond geometry (Å, °) C*g*1, C*g*2, C*g*3 and C*g*4 are the centroids of rings C1*A*–C6*A*, C1*B*–C6*B*, C1*C*–C6*C* and C1*D*–C6*D*, respectively.

*D*—H⋯*A*	*D*—H	H⋯*A*	*D*⋯*A*	*D*—H⋯*A*
O4—H4⋯O1*C*	0.84	1.92	2.762 (3)	177
O3*D*—H3*D*⋯O2*B* ^i^	0.84	1.86	2.695 (2)	172
O2′—H2′⋯O1′^ii^	0.84	1.76	2.532 (9)	151
O2—H2⋯O4*D* ^ii^	0.84	1.97	2.756 (4)	155
C7*D*—H7*D*1⋯O1^ii^	0.98	2.46	3.424 (3)	168
C9*A*—H9*A*1⋯O1	0.99	2.44	3.419 (4)	169
C7*C*—H7*C*2⋯O4*D* ^ii^	0.98	2.63	3.587 (4)	165
C2′—H2*A′*⋯C*g*2	0.98	2.55	3.405 (6)	146
C2′—H2*B′*⋯C*g*3	0.98	2.52	3.457 (8)	159
C2—H2*A*⋯C*g*1	0.98	2.62	3.394 (2)	136
C2—H2*B*⋯C*g*4	0.98	2.94	3.584 (3)	124
C2—H2*C*⋯C*g*3	0.98	2.75	3.694 (4)	163

Symmetry codes: (i) 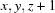; (ii) 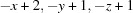.

**Table 2:** Experimental details

Crystal data	
Chemical formula	C_37_H_32_O_10_·2C_2_H_4_O_2_
*M*_r_	756.73
Crystal system, space group	Triclinic, *P* 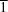
Temperature (K)	190
*a*, *b*, *c* (Å)	11.7576 (7), 11.9561 (8), 14.1979 (9)
α, β, γ (°)	91.710 (1), 105.728 (1), 111.980 (1)
*V* (Å^3^)	1762.12 (19)
*Z*	2
Radiation type	Mo *K*α
μ (mm^−1^)	0.11
Crystal size (mm)	0.10 × 0.09 × 0.07

Data collection	
Diffractometer	Bruker APEXII CCD area-detector
Absorption correction	Multi-scan (*SADABS*; Bruker, 2008 ▸)
*T*_min_, *T*_max_	0.665, 0.746
No. of measured, independent and observed [*I* > 2σ(*I*)] reflections	27938, 10718, 6891
*R*_int_	0.035
(sin θ/λ)_max_ (Å^−1^)	0.717

Refinement	
*R*[*F* ^2^ > 2σ(*F* ^2^)], *wR*(*F* ^2^), *S*	0.067, 0.221, 1.11
No. of reflections	10718
No. of parameters	540
No. of restraints	1
H-atom treatment	H atoms treated by a mixture of independent and constrained refinement
Δρ_max_, Δρ_min_ (e Å^−3^)	1.18, −1.06

Computer programs: *APEX2* and *SAINT* (Bruker, 2008 ▸), *SIR97* (Altomare *et al.*, 1999 ▸), *Mercury* (Macrae *et al.*, 2008 ▸), *WinGX* (Farrugia, 2012 ▸), *PARST* (Nardelli, 1995 ▸), *SHELXL2014* (Sheldrick, 2015 ▸) and *publCIF* (Westrip, 2010 ▸).

## supplementary crystallographic information

### Crystal data

**Table:**

C_37_H_32_O_10_·2C_2_H_4_O_2_	*Z* = 2
*M**_r_* = 756.73	*F*(000) = 796
Triclinic, *P*1	*D*_x_ = 1.426 Mg m^−^^3^
*a* = 11.7576 (7) Å	Mo *K*α radiation, λ = 0.71069 Å
*b* = 11.9561 (8) Å	Cell parameters from 825 reflections
*c* = 14.1979 (9) Å	θ = 1.5–30.7°
α = 91.710 (1)°	µ = 0.11 mm^−^^1^
β = 105.728 (1)°	*T* = 190 K
γ = 111.980 (1)°	Prismatic, colourless
*V* = 1762.12 (19) Å^3^	0.10 × 0.09 × 0.07 mm

### Data collection

**Table:**

Bruker APEXII CCD area-detector diffractometer	10718 independent reflections
Radiation source: fine-focus sealed tube	6891 reflections with *I* > 2σ(*I*)
Graphite monochromator	*R*_int_ = 0.035
ω–scan	θ_max_ = 30.7°, θ_min_ = 1.5°
Absorption correction: multi-scan (SADABS; Bruker, 2008)	*h* = −16→16
*T*_min_ = 0.665, *T*_max_ = 0.746	*k* = −16→16
27938 measured reflections	*l* = −20→20

### Refinement

**Table:**

Refinement on *F*^2^	Primary atom site location: structure-invariant direct methods
Least-squares matrix: full	Secondary atom site location: difference Fourier map
*R*[*F*^2^ > 2σ(*F*^2^)] = 0.067	Hydrogen site location: mixed
*wR*(*F*^2^) = 0.221	H atoms treated by a mixture of independent and constrained refinement
*S* = 1.11	*w* = 1/[σ^2^(*F*_o_^2^) + (0.1037*P*)^2^ + 0.8387*P*] where *P* = (*F*_o_^2^ + 2*F*_c_^2^)/3
10718 reflections	(Δ/σ)_max_ < 0.001
540 parameters	Δρ_max_ = 1.18 e Å^−^^3^
1 restraint	Δρ_min_ = −1.06 e Å^−^^3^

### Special details

**Table:**

Geometry. All esds (except the esd in the dihedral angle between two l.s. planes) are estimated using the full covariance matrix. The cell esds are taken into account individually in the estimation of esds in distances, angles and torsion angles; correlations between esds in cell parameters are only used when they are defined by crystal symmetry. An approximate (isotropic) treatment of cell esds is used for estimating esds involving l.s. planes.

### Fractional atomic coordinates and isotropic or equivalent isotropic displacement parameters (Å^2^)

**Table:**

	*x*	*y*	*z*	*U*_iso_*/*U*_eq_	Occ. (<1)
O1A	0.61841 (17)	0.66131 (14)	0.39569 (12)	0.0307 (3)	
O2A	0.62737 (17)	0.69320 (14)	0.23478 (12)	0.0315 (4)	
O1B	0.74103 (16)	0.49840 (15)	−0.00175 (12)	0.0313 (4)	
O2B	0.81811 (16)	0.34225 (14)	0.00109 (11)	0.0290 (3)	
O1C	0.88379 (15)	0.06566 (14)	0.22558 (11)	0.0286 (3)	
O2C	0.85750 (15)	0.02305 (14)	0.38186 (11)	0.0277 (3)	
O1D	0.71334 (17)	0.18216 (15)	0.61609 (11)	0.0326 (4)	
O2D	0.71907 (16)	0.37445 (15)	0.58604 (12)	0.0337 (4)	
O3D	0.7059 (2)	0.25775 (19)	0.80637 (13)	0.0497 (5)	
H3D	0.7476	0.2875	0.8658	0.075*	
O4D	0.8577 (2)	0.4168 (2)	0.77768 (14)	0.0557 (6)	
C1A	0.4502 (2)	0.32928 (19)	0.36199 (15)	0.0248 (4)	
H1A	0.3770	0.2663	0.3171	0.030*	
C2A	0.4844 (2)	0.44747 (19)	0.34042 (15)	0.0247 (4)	
C3A	0.5888 (2)	0.53913 (19)	0.40881 (16)	0.0252 (4)	
C4A	0.6633 (2)	0.51474 (19)	0.49382 (16)	0.0264 (4)	
C5A	0.6277 (2)	0.3920 (2)	0.50878 (15)	0.0255 (4)	
C6A	0.5183 (2)	0.29861 (18)	0.44650 (15)	0.0230 (4)	
C7A	0.7794 (2)	0.6150 (2)	0.56418 (18)	0.0328 (5)	
H7A1	0.8570	0.6196	0.5483	0.049*	
H7A2	0.7865	0.5979	0.6321	0.049*	
H7A3	0.7703	0.6929	0.5579	0.049*	
C8A	0.4204 (2)	0.4732 (2)	0.24062 (16)	0.0272 (4)	
H8A1	0.4112	0.5517	0.2483	0.033*	
H8A2	0.3335	0.4079	0.2112	0.033*	
C9A	0.6990 (2)	0.7078 (2)	0.33572 (17)	0.0306 (5)	
H9A1	0.7577	0.6650	0.3416	0.037*	
H9A2	0.7524	0.7955	0.3599	0.037*	
C1B	0.4841 (2)	0.3757 (2)	0.11308 (15)	0.0241 (4)	
H1B	0.4148	0.3010	0.1108	0.029*	
C2B	0.5640 (2)	0.3789 (2)	0.05574 (15)	0.0255 (4)	
C3B	0.6641 (2)	0.4898 (2)	0.05990 (15)	0.0266 (4)	
C4B	0.6875 (2)	0.5953 (2)	0.11919 (16)	0.0277 (4)	
C5B	0.6060 (2)	0.58683 (19)	0.17643 (15)	0.0266 (4)	
C6B	0.5029 (2)	0.4789 (2)	0.17391 (15)	0.0247 (4)	
C7B	0.7971 (3)	0.7142 (2)	0.1225 (2)	0.0386 (6)	
H7B1	0.8737	0.7218	0.1759	0.058*	
H7B2	0.7730	0.7822	0.1344	0.058*	
H7B3	0.8158	0.7159	0.0592	0.058*	
C8B	0.5480 (2)	0.2652 (2)	−0.00619 (15)	0.0267 (4)	
H8B1	0.5677	0.2866	−0.0684	0.032*	
H8B2	0.4576	0.2054	−0.0235	0.032*	
C9B	0.8468 (2)	0.4669 (2)	0.03529 (18)	0.0312 (5)	
H9B1	0.8731	0.4800	0.1085	0.037*	
H9B2	0.9201	0.5210	0.0145	0.037*	
C1C	0.5923 (2)	0.11493 (19)	0.10363 (15)	0.0244 (4)	
H1C	0.5037	0.0828	0.0994	0.029*	
C2C	0.6718 (2)	0.06587 (18)	0.16276 (15)	0.0233 (4)	
C3C	0.8018 (2)	0.11562 (19)	0.16907 (15)	0.0245 (4)	
C4C	0.8535 (2)	0.20992 (19)	0.11752 (15)	0.0256 (4)	
C5C	0.7676 (2)	0.25376 (19)	0.05860 (15)	0.0246 (4)	
C6C	0.6373 (2)	0.20898 (19)	0.05054 (14)	0.0243 (4)	
C7C	0.9953 (2)	0.2644 (2)	0.12599 (19)	0.0341 (5)	
H7C1	1.0426	0.2346	0.1802	0.051*	
H7C2	1.0281	0.3535	0.1394	0.051*	
H7C3	1.0070	0.2402	0.0639	0.051*	
C8C	0.6212 (2)	−0.03119 (18)	0.22414 (15)	0.0247 (4)	
H8C1	0.6643	−0.0885	0.2268	0.030*	
H8C2	0.5276	−0.0779	0.1928	0.030*	
C9C	0.9316 (2)	0.1039 (2)	0.33057 (16)	0.0282 (4)	
H9C1	1.0215	0.1107	0.3551	0.034*	
H9C2	0.9322	0.1858	0.3447	0.034*	
C1D	0.5546 (2)	0.05972 (18)	0.35141 (15)	0.0240 (4)	
H1D	0.4756	0.0430	0.3014	0.029*	
C2D	0.5737 (2)	0.11712 (18)	0.44457 (15)	0.0237 (4)	
C3D	0.6879 (2)	0.13624 (19)	0.51763 (15)	0.0249 (4)	
C4D	0.7843 (2)	0.10523 (19)	0.49908 (15)	0.0252 (4)	
C5D	0.7613 (2)	0.05246 (18)	0.40257 (16)	0.0248 (4)	
C6D	0.6453 (2)	0.02588 (18)	0.32814 (15)	0.0233 (4)	
C7D	0.9073 (2)	0.1284 (2)	0.57884 (18)	0.0365 (5)	
H7D1	0.9487	0.2149	0.6071	0.055*	
H7D2	0.9651	0.1067	0.5508	0.055*	
H7D3	0.8887	0.0788	0.6308	0.055*	
C8D	0.4749 (2)	0.16357 (19)	0.45784 (16)	0.0250 (4)	
H8D1	0.4644	0.1525	0.5243	0.030*	
H8D2	0.3907	0.1163	0.4078	0.030*	
C9D	0.6807 (2)	0.2806 (2)	0.63988 (16)	0.0307 (5)	
C10D	0.7613 (3)	0.3273 (2)	0.74892 (17)	0.0342 (5)	
O1'	0.9221 (6)	0.5528 (5)	0.3967 (5)	0.0456 (16)	0.344 (4)
O2'	0.9045 (7)	0.3631 (6)	0.4379 (5)	0.065 (2)	0.344 (4)
H2'	0.9756	0.4048	0.4801	0.097*	0.344 (4)
C1'	0.8681 (10)	0.4324 (9)	0.3810 (7)	0.048 (2)	0.344 (4)
C2'	0.754 (2)	0.375 (2)	0.2966 (12)	0.052 (4)	0.344 (4)
H2A'	0.7373	0.4382	0.2595	0.078*	0.344 (4)
H2B'	0.7682	0.3191	0.2537	0.078*	0.344 (4)
H2C'	0.6806	0.3302	0.3193	0.078*	0.344 (4)
O1	0.9054 (4)	0.5722 (3)	0.3241 (4)	0.0692 (10)	0.656 (4)
O2	0.9718 (3)	0.4287 (3)	0.3080 (4)	0.0692 (10)	0.656 (4)
H2	1.0335	0.4875	0.2996	0.104*	0.656 (4)
C1	0.8883 (4)	0.4674 (3)	0.3251 (3)	0.0339 (9)	0.656 (4)
C2	0.7677 (9)	0.3670 (9)	0.3298 (6)	0.0424 (18)	0.656 (4)
H2A	0.6993	0.3974	0.3210	0.064*	0.656 (4)
H2B	0.7835	0.3387	0.3942	0.064*	0.656 (4)
H2C	0.7412	0.2992	0.2772	0.064*	0.656 (4)
O3	1.1809 (2)	0.0884 (3)	0.2430 (2)	0.0782 (8)	
O4	1.0071 (2)	−0.0252 (2)	0.12312 (17)	0.0552 (6)	
H4	0.9719	0.0042	0.1553	0.083*	
C3	1.1327 (3)	0.0238 (3)	0.1649 (2)	0.0479 (7)	
C4	1.2037 (4)	−0.0085 (5)	0.1052 (3)	0.0868 (14)	
H4A	1.1692	0.0002	0.0360	0.130*	
H4B	1.1941	−0.0931	0.1093	0.130*	
H4C	1.2951	0.0458	0.1304	0.130*	
H9D	0.596 (3)	0.271 (2)	0.6529 (14)	0.066 (10)*	

### Atomic displacement parameters (Å^2^)

**Table:**

	*U*^11^	*U*^22^	*U*^33^	*U*^12^	*U*^13^	*U*^23^
O1A	0.0451 (9)	0.0222 (7)	0.0330 (8)	0.0172 (7)	0.0192 (7)	0.0072 (6)
O2A	0.0485 (10)	0.0248 (8)	0.0304 (8)	0.0206 (7)	0.0170 (7)	0.0084 (6)
O1B	0.0397 (9)	0.0378 (9)	0.0299 (8)	0.0239 (7)	0.0182 (7)	0.0145 (7)
O2B	0.0413 (9)	0.0316 (8)	0.0255 (7)	0.0207 (7)	0.0181 (7)	0.0112 (6)
O1C	0.0335 (8)	0.0308 (8)	0.0311 (8)	0.0198 (7)	0.0141 (7)	0.0091 (6)
O2C	0.0335 (8)	0.0264 (8)	0.0322 (8)	0.0178 (7)	0.0150 (7)	0.0098 (6)
O1D	0.0508 (10)	0.0345 (9)	0.0207 (7)	0.0260 (8)	0.0105 (7)	0.0058 (6)
O2D	0.0340 (9)	0.0319 (9)	0.0318 (8)	0.0119 (7)	0.0058 (7)	0.0100 (7)
O3D	0.0628 (13)	0.0514 (12)	0.0236 (8)	0.0115 (10)	0.0116 (8)	0.0074 (8)
O4D	0.0542 (12)	0.0589 (13)	0.0321 (10)	0.0013 (10)	0.0096 (9)	0.0044 (9)
C1A	0.0258 (10)	0.0252 (10)	0.0262 (10)	0.0111 (8)	0.0111 (8)	0.0035 (8)
C2A	0.0286 (10)	0.0274 (10)	0.0256 (10)	0.0147 (9)	0.0146 (8)	0.0062 (8)
C3A	0.0329 (11)	0.0211 (9)	0.0269 (10)	0.0124 (8)	0.0150 (8)	0.0035 (8)
C4A	0.0290 (10)	0.0254 (10)	0.0274 (10)	0.0109 (8)	0.0128 (8)	0.0027 (8)
C5A	0.0281 (10)	0.0267 (10)	0.0246 (10)	0.0124 (8)	0.0102 (8)	0.0055 (8)
C6A	0.0269 (10)	0.0221 (9)	0.0255 (9)	0.0109 (8)	0.0149 (8)	0.0052 (7)
C7A	0.0309 (11)	0.0281 (11)	0.0348 (12)	0.0086 (9)	0.0079 (9)	0.0003 (9)
C8A	0.0300 (11)	0.0300 (11)	0.0278 (10)	0.0173 (9)	0.0108 (8)	0.0053 (8)
C9A	0.0404 (13)	0.0224 (10)	0.0312 (11)	0.0118 (9)	0.0152 (10)	0.0063 (8)
C1B	0.0248 (10)	0.0262 (10)	0.0218 (9)	0.0128 (8)	0.0038 (8)	0.0038 (7)
C2B	0.0307 (11)	0.0300 (11)	0.0193 (9)	0.0175 (9)	0.0049 (8)	0.0054 (8)
C3B	0.0325 (11)	0.0328 (11)	0.0234 (10)	0.0195 (9)	0.0121 (8)	0.0105 (8)
C4B	0.0339 (11)	0.0276 (10)	0.0263 (10)	0.0156 (9)	0.0107 (9)	0.0124 (8)
C5B	0.0373 (12)	0.0238 (10)	0.0235 (10)	0.0175 (9)	0.0088 (9)	0.0058 (8)
C6B	0.0281 (10)	0.0297 (10)	0.0221 (9)	0.0174 (9)	0.0078 (8)	0.0068 (8)
C7B	0.0436 (14)	0.0295 (12)	0.0452 (14)	0.0115 (11)	0.0210 (12)	0.0109 (10)
C8B	0.0297 (11)	0.0331 (11)	0.0196 (9)	0.0163 (9)	0.0059 (8)	0.0023 (8)
C9B	0.0336 (12)	0.0310 (11)	0.0358 (12)	0.0155 (10)	0.0169 (10)	0.0109 (9)
C1C	0.0263 (10)	0.0244 (10)	0.0212 (9)	0.0099 (8)	0.0059 (8)	−0.0027 (7)
C2C	0.0292 (10)	0.0194 (9)	0.0214 (9)	0.0093 (8)	0.0089 (8)	0.0000 (7)
C3C	0.0311 (11)	0.0244 (10)	0.0238 (9)	0.0157 (8)	0.0103 (8)	0.0046 (8)
C4C	0.0299 (11)	0.0266 (10)	0.0240 (10)	0.0125 (9)	0.0119 (8)	0.0038 (8)
C5C	0.0329 (11)	0.0252 (10)	0.0201 (9)	0.0139 (9)	0.0116 (8)	0.0036 (7)
C6C	0.0312 (11)	0.0260 (10)	0.0170 (9)	0.0141 (8)	0.0058 (8)	−0.0016 (7)
C7C	0.0318 (12)	0.0378 (13)	0.0394 (13)	0.0169 (10)	0.0161 (10)	0.0129 (10)
C8C	0.0302 (11)	0.0175 (9)	0.0259 (10)	0.0083 (8)	0.0101 (8)	0.0007 (7)
C9C	0.0275 (11)	0.0277 (11)	0.0324 (11)	0.0137 (9)	0.0096 (9)	0.0069 (9)
C1D	0.0253 (10)	0.0185 (9)	0.0260 (10)	0.0062 (8)	0.0078 (8)	0.0043 (7)
C2D	0.0292 (10)	0.0173 (9)	0.0268 (10)	0.0081 (8)	0.0133 (8)	0.0056 (7)
C3D	0.0346 (11)	0.0213 (9)	0.0214 (9)	0.0117 (8)	0.0114 (8)	0.0049 (7)
C4D	0.0293 (10)	0.0225 (10)	0.0249 (10)	0.0117 (8)	0.0073 (8)	0.0066 (8)
C5D	0.0294 (10)	0.0196 (9)	0.0283 (10)	0.0111 (8)	0.0112 (8)	0.0057 (8)
C6D	0.0290 (10)	0.0179 (9)	0.0239 (9)	0.0080 (8)	0.0110 (8)	0.0045 (7)
C7D	0.0373 (13)	0.0417 (14)	0.0312 (12)	0.0211 (11)	0.0037 (10)	0.0044 (10)
C8D	0.0275 (10)	0.0225 (10)	0.0281 (10)	0.0096 (8)	0.0137 (8)	0.0053 (8)
C9D	0.0409 (13)	0.0293 (11)	0.0246 (10)	0.0149 (10)	0.0126 (9)	0.0045 (8)
C10D	0.0421 (13)	0.0364 (13)	0.0277 (11)	0.0186 (11)	0.0118 (10)	0.0042 (9)
O1'	0.062 (4)	0.028 (3)	0.045 (3)	0.017 (3)	0.015 (3)	−0.002 (2)
O2'	0.069 (5)	0.046 (4)	0.067 (4)	0.025 (3)	−0.002 (3)	0.004 (3)
C1'	0.059 (6)	0.057 (5)	0.051 (5)	0.044 (5)	0.025 (5)	0.017 (4)
C2'	0.069 (8)	0.044 (6)	0.054 (10)	0.029 (6)	0.024 (8)	0.011 (7)
O1	0.0467 (14)	0.0397 (13)	0.128 (3)	0.0134 (11)	0.0420 (16)	0.0124 (15)
O2	0.0467 (14)	0.0397 (13)	0.128 (3)	0.0134 (11)	0.0420 (16)	0.0124 (15)
C1	0.0356 (19)	0.0272 (18)	0.044 (2)	0.0150 (15)	0.0149 (16)	0.0094 (16)
C2	0.034 (3)	0.037 (3)	0.064 (6)	0.013 (2)	0.027 (4)	0.009 (4)
O3	0.0506 (14)	0.091 (2)	0.0718 (17)	0.0179 (13)	0.0029 (12)	−0.0209 (15)
O4	0.0396 (11)	0.0559 (13)	0.0631 (14)	0.0132 (10)	0.0152 (10)	−0.0105 (10)
C3	0.0386 (14)	0.0442 (16)	0.0551 (17)	0.0129 (12)	0.0111 (13)	0.0005 (13)
C4	0.059 (2)	0.123 (4)	0.084 (3)	0.044 (2)	0.023 (2)	−0.011 (3)

### Geometric parameters (Å, º)

**Table:**

O1A—C3A	1.400 (3)	C1C—C6C	1.390 (3)
O1A—C9A	1.415 (3)	C1C—C2C	1.392 (3)
O2A—C5B	1.397 (3)	C1C—H1C	0.9500
O2A—C9A	1.419 (3)	C2C—C3C	1.393 (3)
O1B—C3B	1.400 (3)	C2C—C8C	1.512 (3)
O1B—C9B	1.405 (3)	C3C—C4C	1.398 (3)
O2B—C5C	1.408 (2)	C4C—C5C	1.399 (3)
O2B—C9B	1.436 (3)	C4C—C7C	1.514 (3)
O1C—C3C	1.403 (2)	C5C—C6C	1.391 (3)
O1C—C9C	1.435 (3)	C7C—H7C1	0.9800
O2C—C5D	1.400 (3)	C7C—H7C2	0.9800
O2C—C9C	1.407 (3)	C7C—H7C3	0.9800
O1D—C3D	1.396 (2)	C8C—C6D	1.515 (3)
O1D—C9D	1.425 (3)	C8C—H8C1	0.9900
O2D—C9D	1.381 (3)	C8C—H8C2	0.9900
O2D—C5A	1.395 (3)	C9C—H9C1	0.9900
O3D—C10D	1.305 (3)	C9C—H9C2	0.9900
O3D—H3D	0.8400	C1D—C6D	1.383 (3)
O4D—C10D	1.188 (3)	C1D—C2D	1.393 (3)
C1A—C2A	1.385 (3)	C1D—H1D	0.9500
C1A—C6A	1.393 (3)	C2D—C3D	1.390 (3)
C1A—H1A	0.9500	C2D—C8D	1.513 (3)
C2A—C3A	1.391 (3)	C3D—C4D	1.400 (3)
C2A—C8A	1.513 (3)	C4D—C5D	1.403 (3)
C3A—C4A	1.396 (3)	C4D—C7D	1.498 (3)
C4A—C5A	1.408 (3)	C5D—C6D	1.397 (3)
C4A—C7A	1.504 (3)	C7D—H7D1	0.9800
C5A—C6A	1.390 (3)	C7D—H7D2	0.9800
C6A—C8D	1.529 (3)	C7D—H7D3	0.9800
C7A—H7A1	0.9800	C8D—H8D1	0.9900
C7A—H7A2	0.9800	C8D—H8D2	0.9900
C7A—H7A3	0.9800	C9D—C10D	1.537 (3)
C8A—C6B	1.514 (3)	C9D—H9D	1.03 (4)
C8A—H8A1	0.9900	O1'—C1'	1.321 (11)
C8A—H8A2	0.9900	O2'—C1'	1.281 (11)
C9A—H9A1	0.9900	O2'—H2'	0.8400
C9A—H9A2	0.9900	C1'—C2'	1.45 (2)
C1B—C2B	1.391 (3)	C2'—H2A'	0.9800
C1B—C6B	1.395 (3)	C2'—H2B'	0.9800
C1B—H1B	0.9500	C2'—H2C'	0.9800
C2B—C3B	1.391 (3)	O1—C1	1.193 (5)
C2B—C8B	1.518 (3)	O2—C1	1.304 (5)
C3B—C4B	1.388 (3)	O2—H2	0.8400
C4B—C5B	1.394 (3)	C1—C2	1.496 (10)
C4B—C7B	1.509 (3)	C2—H2A	0.9800
C5B—C6B	1.392 (3)	C2—H2B	0.9800
C7B—H7B1	0.9800	C2—H2C	0.9800
C7B—H7B2	0.9800	O3—C3	1.196 (4)
C7B—H7B3	0.9800	O4—C3	1.316 (3)
C8B—C6C	1.519 (3)	O4—H4	0.8400
C8B—H8B1	0.9900	C3—C4	1.473 (5)
C8B—H8B2	0.9900	C4—H4A	0.9800
C9B—H9B1	0.9900	C4—H4B	0.9800
C9B—H9B2	0.9900	C4—H4C	0.9800
			
C3A—O1A—C9A	116.28 (16)	C6C—C5C—C4C	123.35 (19)
C5B—O2A—C9A	115.63 (16)	C6C—C5C—O2B	120.26 (18)
C3B—O1B—C9B	115.94 (17)	C4C—C5C—O2B	116.26 (18)
C5C—O2B—C9B	117.48 (16)	C1C—C6C—C5C	117.03 (19)
C3C—O1C—C9C	117.46 (16)	C1C—C6C—C8B	120.16 (19)
C5D—O2C—C9C	115.82 (16)	C5C—C6C—C8B	122.62 (19)
C3D—O1D—C9D	120.65 (16)	C4C—C7C—H7C1	109.5
C9D—O2D—C5A	119.84 (18)	C4C—C7C—H7C2	109.5
C10D—O3D—H3D	109.5	H7C1—C7C—H7C2	109.5
C2A—C1A—C6A	123.0 (2)	C4C—C7C—H7C3	109.5
C2A—C1A—H1A	118.5	H7C1—C7C—H7C3	109.5
C6A—C1A—H1A	118.5	H7C2—C7C—H7C3	109.5
C1A—C2A—C3A	117.7 (2)	C2C—C8C—C6D	110.61 (16)
C1A—C2A—C8A	120.8 (2)	C2C—C8C—H8C1	109.5
C3A—C2A—C8A	121.14 (19)	C6D—C8C—H8C1	109.5
C2A—C3A—C4A	122.38 (19)	C2C—C8C—H8C2	109.5
C2A—C3A—O1A	119.78 (19)	C6D—C8C—H8C2	109.5
C4A—C3A—O1A	117.78 (19)	H8C1—C8C—H8C2	108.1
C3A—C4A—C5A	117.06 (19)	O2C—C9C—O1C	112.73 (18)
C3A—C4A—C7A	121.3 (2)	O2C—C9C—H9C1	109.0
C5A—C4A—C7A	121.6 (2)	O1C—C9C—H9C1	109.0
C6A—C5A—O2D	124.55 (19)	O2C—C9C—H9C2	109.0
C6A—C5A—C4A	122.5 (2)	O1C—C9C—H9C2	109.0
O2D—C5A—C4A	112.76 (19)	H9C1—C9C—H9C2	107.8
C5A—C6A—C1A	117.13 (19)	C6D—C1D—C2D	123.2 (2)
C5A—C6A—C8D	125.11 (19)	C6D—C1D—H1D	118.4
C1A—C6A—C8D	117.45 (19)	C2D—C1D—H1D	118.4
C4A—C7A—H7A1	109.5	C3D—C2D—C1D	117.39 (19)
C4A—C7A—H7A2	109.5	C3D—C2D—C8D	124.06 (19)
H7A1—C7A—H7A2	109.5	C1D—C2D—C8D	118.36 (19)
C4A—C7A—H7A3	109.5	C2D—C3D—O1D	122.94 (19)
H7A1—C7A—H7A3	109.5	C2D—C3D—C4D	122.42 (19)
H7A2—C7A—H7A3	109.5	O1D—C3D—C4D	114.57 (19)
C2A—C8A—C6B	108.64 (17)	C3D—C4D—C5D	117.27 (19)
C2A—C8A—H8A1	110.0	C3D—C4D—C7D	121.3 (2)
C6B—C8A—H8A1	110.0	C5D—C4D—C7D	121.4 (2)
C2A—C8A—H8A2	110.0	C6D—C5D—O2C	119.52 (18)
C6B—C8A—H8A2	110.0	C6D—C5D—C4D	122.23 (19)
H8A1—C8A—H8A2	108.3	O2C—C5D—C4D	118.23 (19)
O1A—C9A—O2A	112.08 (19)	C1D—C6D—C5D	117.37 (19)
O1A—C9A—H9A1	109.2	C1D—C6D—C8C	120.65 (19)
O2A—C9A—H9A1	109.2	C5D—C6D—C8C	121.89 (19)
O1A—C9A—H9A2	109.2	C4D—C7D—H7D1	109.5
O2A—C9A—H9A2	109.2	C4D—C7D—H7D2	109.5
H9A1—C9A—H9A2	107.9	H7D1—C7D—H7D2	109.5
C2B—C1B—C6B	122.0 (2)	C4D—C7D—H7D3	109.5
C2B—C1B—H1B	119.0	H7D1—C7D—H7D3	109.5
C6B—C1B—H1B	119.0	H7D2—C7D—H7D3	109.5
C1B—C2B—C3B	117.76 (19)	C2D—C8D—C6A	109.90 (17)
C1B—C2B—C8B	122.0 (2)	C2D—C8D—H8D1	109.7
C3B—C2B—C8B	120.20 (19)	C6A—C8D—H8D1	109.7
C4B—C3B—C2B	122.8 (2)	C2D—C8D—H8D2	109.7
C4B—C3B—O1B	117.8 (2)	C6A—C8D—H8D2	109.7
C2B—C3B—O1B	119.35 (19)	H8D1—C8D—H8D2	108.2
C3B—C4B—C5B	117.2 (2)	O2D—C9D—O1D	112.32 (19)
C3B—C4B—C7B	121.6 (2)	O2D—C9D—C10D	107.95 (19)
C5B—C4B—C7B	121.2 (2)	O1D—C9D—C10D	103.03 (18)
C6B—C5B—C4B	122.5 (2)	O2D—C9D—H9D	111.3 (18)
C6B—C5B—O2A	119.86 (19)	O1D—C9D—H9D	125.1 (15)
C4B—C5B—O2A	117.6 (2)	C10D—C9D—H9D	93.6 (7)
C5B—C6B—C1B	117.69 (19)	O4D—C10D—O3D	124.4 (2)
C5B—C6B—C8A	120.47 (19)	O4D—C10D—C9D	125.1 (2)
C1B—C6B—C8A	121.7 (2)	O3D—C10D—C9D	110.5 (2)
C4B—C7B—H7B1	109.5	C1'—O2'—H2'	109.5
C4B—C7B—H7B2	109.5	O2'—C1'—O1'	124.9 (9)
H7B1—C7B—H7B2	109.5	O2'—C1'—C2'	117.8 (11)
C4B—C7B—H7B3	109.5	O1'—C1'—C2'	117.2 (11)
H7B1—C7B—H7B3	109.5	C1'—C2'—H2A'	109.5
H7B2—C7B—H7B3	109.5	C1'—C2'—H2B'	109.5
C2B—C8B—C6C	110.35 (16)	H2A'—C2'—H2B'	109.5
C2B—C8B—H8B1	109.6	C1'—C2'—H2C'	109.5
C6C—C8B—H8B1	109.6	H2A'—C2'—H2C'	109.5
C2B—C8B—H8B2	109.6	H2B'—C2'—H2C'	109.5
C6C—C8B—H8B2	109.6	C1—O2—H2	109.5
H8B1—C8B—H8B2	108.1	O1—C1—O2	120.0 (4)
O1B—C9B—O2B	112.21 (19)	O1—C1—C2	125.9 (5)
O1B—C9B—H9B1	109.2	O2—C1—C2	113.4 (5)
O2B—C9B—H9B1	109.2	C1—C2—H2A	109.5
O1B—C9B—H9B2	109.2	C1—C2—H2B	109.5
O2B—C9B—H9B2	109.2	H2A—C2—H2B	109.5
H9B1—C9B—H9B2	107.9	C1—C2—H2C	109.5
C6C—C1C—C2C	122.8 (2)	H2A—C2—H2C	109.5
C6C—C1C—H1C	118.6	H2B—C2—H2C	109.5
C2C—C1C—H1C	118.6	C3—O4—H4	109.5
C1C—C2C—C3C	117.62 (19)	O3—C3—O4	121.7 (3)
C1C—C2C—C8C	121.21 (19)	O3—C3—C4	125.2 (3)
C3C—C2C—C8C	121.01 (18)	O4—C3—C4	113.1 (3)
C2C—C3C—C4C	122.69 (19)	C3—C4—H4A	109.5
C2C—C3C—O1C	119.02 (18)	C3—C4—H4B	109.5
C4C—C3C—O1C	118.22 (19)	H4A—C4—H4B	109.5
C3C—C4C—C5C	116.55 (19)	C3—C4—H4C	109.5
C3C—C4C—C7C	122.21 (19)	H4A—C4—H4C	109.5
C5C—C4C—C7C	121.22 (19)	H4B—C4—H4C	109.5

### Hydrogen-bond geometry (Å, º)

C*g*1, C*g*2, C*g*3 and C*g*4 are the centroids of rings C1*A*–C6*A*, C1*B*–C6*B*, C1*C*–C6*C* and C1*D*–C6*D*, respectively.

**Table:**

*D*—H···*A*	*D*—H	H···*A*	*D*···*A*	*D*—H···*A*
O4—H4···O1*C*	0.84	1.92	2.762 (3)	177
O3*D*—H3*D*···O2*B*^i^	0.84	1.86	2.695 (2)	172
O2′—H2′···O1′^ii^	0.84	1.76	2.532 (9)	151
O2—H2···O4*D*^ii^	0.84	1.97	2.756 (4)	155
C7*D*—H7*D*1···O1^ii^	0.98	2.46	3.424 (3)	168
C9*A*—H9*A*1···O1	0.99	2.44	3.419 (4)	169
C7*C*—H7*C*2···O4*D*^ii^	0.98	2.63	3.587 (4)	165
C2′—H2*A′*···C*g*2	0.98	2.55	3.405 (6)	146
C2′—H2*B′*···C*g*3	0.98	2.52	3.457 (8)	159
C2—H2*A*···C*g*1	0.98	2.62	3.394 (2)	136
C2—H2*B*···C*g*4	0.98	2.94	3.584 (3)	124
C2—H2*C*···C*g*3	0.98	2.75	3.694 (4)	163

Symmetry codes: (i) *x*, *y*, *z*+1; (ii) −*x*+2, −*y*+1, −*z*+1.
